# A real-time monitoring platform of myogenesis regulators using double fluorescent labeling

**DOI:** 10.1371/journal.pone.0192654

**Published:** 2018-02-14

**Authors:** Etai Sapoznik, Guoguang Niu, Yu Zhou, Peter M. Prim, Tracy L. Criswell, Shay Soker

**Affiliations:** Wake Forest Institute for Regenerative Medicine, Winston Salem, North Carolina, United States of America; University of Minnesota Medical Center, UNITED STATES

## Abstract

Real-time, quantitative measurement of muscle progenitor cell (myoblast) differentiation is an important tool for skeletal muscle research and identification of drugs that support skeletal muscle regeneration. While most quantitative tools rely on sacrificial approach, we developed a double fluorescent tagging approach, which allows for dynamic monitoring of myoblast differentiation through assessment of fusion index and nuclei count. Fluorescent tagging of both the cell cytoplasm and nucleus enables monitoring of cell fusion and the formation of new myotube fibers, similar to immunostaining results. This labeling approach allowed monitoring the effects of *Myf5* overexpression, TNFα, and Wnt agonist on myoblast differentiation. It also enabled testing the effects of surface coating on the fusion levels of scaffold-seeded myoblasts. The double fluorescent labeling of myoblasts is a promising technique to visualize even minor changes in myogenesis of myoblasts in order to support applications such as tissue engineering and drug screening.

## Introduction

Live cell imaging can provide important information for regenerative medicine and disease modeling including intermediate steps of a dynamic biological process and elucidating the effects of specific factors on cell phenotype [1]. Skeletal muscle regeneration is an example of the dynamic processes of muscle progenitor cell differentiation and tissue development [2]. Under given conditions muscle satellite cells undergo multiple molecular steps during myogenic differentiation from proliferating myoblasts to muscle fibers [3,4]. Understanding this process is important to determine the potential for efficient regeneration [5] and assists in predicting subsequent muscle function [6]. Myoblast cell models have been used to better understand cell fusion and fiber forming processes, and they created a preliminary platform for testing compounds to treat muscle diseases [6–8]. For example, C2C12, an immortalized murine myoblast cell line, has been extensively used to demonstrate the effects of different compounds on myoblast fusion during drug screening ([9];Hirasaka, 2013 #338;Willkomm, 2014 #82). Since C2C12 cells are different from primary human myoblasts [10,11], lead compounds are finally tested on primary human myoblasts.

Several factors influence myoblast differentiation including surface-substrate and soluble signals. First, physical properties of biomaterials used for tissue engineering, such as stiffness and architecture, play an important role in regulating myoblast phenotype and function [12–14]. Thick scaffolds and 3D architecture have been considered for larger volume muscle regeneration (augmentation) and recapitulating the muscle stem cell niche *in vitro*. Second, soluble factors and insoluble proteins such as integrins trigger signaling pathways that induce the expression of myogenic transcription factors such as MYF5, MYOD, and MYOG, and play a central role in myoblast differentiation [15–17]. To better understand the roles of these factors, loss of function and over-expression techniques, using small molecule activators and inhibitors of various signaling pathways, are commonly used.

Since myoblast differentiation alters morphology from individual cells to myofibers, it is an attractive model for studying the impact of cell signaling on tissue development [18]. The common assessment methods of myoblast differentiation and fusion into myofibers are sacrificial, including immunocytochemistry (ICC), proteomics [19] and genomics [20,21]. ICC allows for quantification of the fusion index, which is defined as the percentage of myoblast nuclei that have fused into fibers in a single time point [22]. Fusion indices are the main tool for assessment of the differentiation potential of myoblasts [23]. Newer live cell imaging tools allow assessment of myoblast differentiation in real-time. Fluorescence microscopy have higher spatial resolution compared with medical tomography and bioluminescence but have lower image penetration depth [24–29]. Optical imaging tools can focus on individual cells, however the imaging depth is typically limited and additional manipulations of the sample are needed such as intravital microscopy [30,31]. Single cell resolution via fluorescent tagging can provide high, single cell resolution and specificity to allow monitoring of specific cell populations. However, high throughput analysis for myoblast differentiation may require a more distinct measure that can be achieved by immunostaining.

In the work presented here, the nuclei and cytoplasm of myoblast were labeled individually with fluorescent tags to better quantify cell fusion and myofiber formation. Labeling combinations were evaluated for image contrast in order to support visualization of cells seeded onto scaffolds and for high throughput imaging capabilities. Double-labeled cell images were compared with standard immunostaining to determine the applicability of an automated image analysis technology. We then used the double fluorescent labeling approach to determine the effects of MYF5 overexpression, TNFα, Wnt pathway agonist, and surface coating on myoblast fusion. Our data suggest that the double labeling technique is a valuable quantitation tool for multiple applications including tissue engineering, disease modeling, and drug screening.

## Methods

### Cell culture and cell labeling with lentivirus

C2C12 cells (mouse myoblasts, ATCC CRL-1772) were cultured on gelatin-coated dishes in growth media, comprised of Dulbecco’s modified eagle medium with 4.5 g/L glucose (DMEM, Thermo Scientific, Waltham, MA) with 20% FBS, 1% penicillin-streptomycin (P/S), and maintained in humidified 5% CO_2_ incubator at 37°C. For each experiment, cells were used at the same batch and within 10 passages. Cells were grown in growth media for about 2–3 days until they reached 90–100% confluence, and then the media was changed to differentiation media containing DMEM and 2% horse serum. The differentiation media media was replaced every 2–3 days. Cells were transduced with lentiviral system to express the fluorescent proteins GFP and mCherry in the cytoplasm and the nucleus. Vectors used include pCDH-GFP (pCDH-null GFP-Puro-System biosciences CD513B-1), mCherry [32] (Addgene plasmid # 31845), H2B-GFP [33] (Addgene plasmid # 25999), H2B mCherry [33] (Addgene plasmid # 21217), Doxycycline-inducible lentivirus hMYF5 overexpression pINDUCER20-FLAG-h*MYF5* (#801) (Addgene plasmid # 78334). Two fluorescent double labeling combinations were used: (1) cytoplasmic GFP (cGFP)+nuclear mCherry (nmCherry) and (2) cytoplasmic mCherry(cmCherry)+nuclear GFP(nGFP). Most experiments used the first combination of cGFP+nmCerry. Fluorescent cells were sorted with FACSAria™II (BD Biosciences) to isolate the highest expressing cells for the two fluorophores.

### Drug treatments

Induction of MYF5 expression from the “Tet-on” promoter was performed with 2μg/ml doxycycline. In some experiments, cells were exposed to 1μM of the Wnt agoinst, 6-bromoindirubin-3'-oxime (BIO, STEMCELL) or to 20 ng/ml recombinant mouse TNFα (aa 80–235, R&D systems Minneapolis, MN). Different drug exposure periods included: no drug (ND), continuous exposure (days 3–10), early exposure during days 3–5 from initiation of differentiation, and late exposure during days 7–9 from initiation of differentiation (Table 1).

**Table 1:** Schedule of media changes.

	Day	1	2	3	4	5	6	7	8	9	10
Growth media	No drug										
Differentiation media	Days 3–5										
Differentiation media +drug	Days 7–9										
	Day 3–10										

### Immunostaining

For immunostaining, cells were fixed with 4% paraformaldehyde (PFA) diluted with PBS for 15 minutes followed by permeabilization in 0.1% Triton-X100/PBS for 5 minutes. Samples were washed in PBS and covered with protein block (Dako X0909) for 30 minutes. After another PBS wash, cells were incubated with the primary antibody diluted in antibody diluent (Dako S3022) for 1 hour. Following another PBS wash, cells were incubated in secondary antibody for 45 minutes. The cells were incubated in DAPI (1:1000) dilution prior to imaging. Antibody used was anti myosin heavy chain (MYH-MF20- developmental studies hybridoma bank–DSHB, IA).

### Epifluorescent imaging

Imaging was performed using two different microscopes. For the initial assessment of contrast, cells were imaged using an inverted fluorescent microscope (Zeiss M1 imager). For cell fusion analyses, cells were imaged in 96-well plates (μclear black cellstar, Greiner bio-one) using an automated fluorescent microscope (GE IN Cell 2000, GE Healthcare Life Sciences, Pittsburgh, PA). Images were taken in one focal plane in the same spatial fields daily, then processed and analyzed on selected days using MATLAB. For live cell dynamic imaging, cells were monitored in the incubator for 10 days using the IncuCyte™ FLR and ZOOM® Live Cell Imaging System.

### Scaffold fabrication

Scaffolds were fabricated from a solution composed of Poly(epsilon-caprolactone) (PCL; Lactel Absorbable Polymers, Pelham, AL) and collagen (Collagen type I, derived from calf skin, Elastin Products Co. Owensville, MO, USA) with a weight ratio of 7:3, respectively. The total amount of the polymer-collagen blend was 7.5 wt% in 1,1,1,3,3,3-hexafluoro-2-propanol (HFP) solution (Sigma Chemical Co. St Louis, MO, USA). Electrospinning was performed as described before [34]. Briefly, the blended solution was ejected through a blunt needle via an electrostatic field (10 kV) and collected on a stainless steel mandrel (35 mm diameter and 30 mm long). The resulting scaffold sheet was then punched to create circular pieces used for cell seeding experiments. Fabrication of collagen film was performed following scaffold fabrication by creating a layer of collagen gel, which was later dried. Following the manufacturer’s instructions for collagen Type I gelation (Corning Incorporated, NY), rat tail collagen Type I (100 mg/ml) was mixed with 10X phosphate buffered saline (10X PBS), 1N NaOH, and dH2O. The mix was added to the surface of the scaffold followed by 1 hour in 37°C for gelation. Subsequently the gel was dried for another 4 hours. Scaffolds were sterilized by washing in 70% ethanol and followed by multiple washes in sterile PBS.

### Image analysis

Image organization, processing, and analysis were performed in MATLAB (Mathworks). A custom-made algorithm was developed for end-user analysis that included the following steps:

1. Choose experimental condition to analyze.
2. Images from different time points appear for analysis as the user can move between fields of view in wells in case of out-of-focus images, and a total of 3 wells are presented with 4–5 time points.
3. At each time point, the user marks larger cell bodies in the cytoplasm channel and uses a semi-automated tool to identify all cell nuclei in the nucleus channel.
4. Based on nuclei positions and fiber masks, the code calculates fusion index and nuclei count. Multinucleated fibers were considered fused with 3 or more nuclei per fiber; 2 nuclei can either be fused or dividing cells.

In the assessment of labeling contrast, the contrast was assessed for each object in the image based on user based marking of object and background regions.

$Contrast\_j=\frac{S_{j}-bgd}{bgd}$, where *Contrast_j* is the contrast of object j and $S_{j}=\frac{\sum_{n=1}^{N}P_{n}}{N}$ is the mean pixel value in the object. $bgd=\frac{\sum_{m=1}^{M}B_{m}}{M}$ is the mean value of all the pixels in the user-defined background region.

### Statistical analysis

Results are shown as mean ± standard deviation, and statistical analyses were performed using Graphpad Prism v5 (Graphpad Software Inc, La Jolla, CA, USA). Unless otherwise stated, tests of statistical significance (α = 0.05) were performed as one-way ANOVAs with Bonferroni adjusted multiple paired comparisons, and there were 3 replicates per unique test condition.

## Results

### Fluorescent double labeling of myoblasts

**Fig 1:**
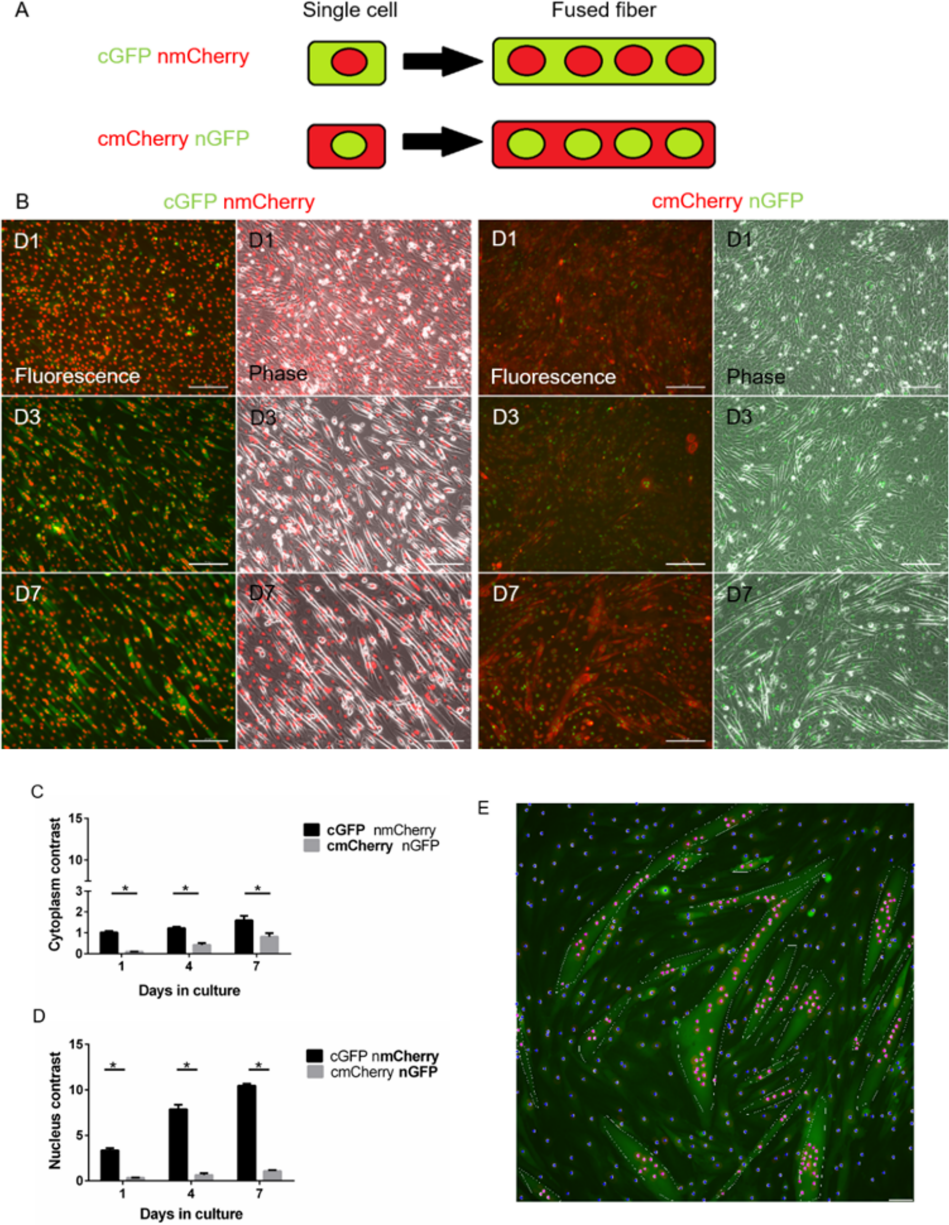
Double fluorescent labeling. (A) Diagram of cGFP+nmCherry and cmCherry+nGFP–. (B) Phase and fluorescent images for the different labeling combinations, as indicated (scale bar 200 **μ**m) (C, D) Contrast comparison between labels for cytoplasm and nucleus. * p < 0.001 on each day (E) Marking of fiber and nuclei for fusion index measurements in cGFP+nmCherry cells, white outline for myotubes, magenta spots for nuclei inside of fibers, and blue spots for nuclei outside of fibers (scale bar 50 **μ**m).

C2C12 is an immortalized murine myoblast cell line that has been extensively used to study regulation of myogenesis *in vitro* [35–37], and was chosen as a model system to express recombinant fluorescent proteins for the current study. C2C12 cells were transduced with lentiviruses encoding for fluorescent proteins localized to the cytoplasm and nucleus to create two combinations: (1) cytoplasmic GFP (cGFP)+nuclear mCherry (nmChery) and (2) cytoplasmic mCherry (cmCherry)+nuclear GFP (nGFP) (Fig 1A). Both labeling combinations showed a progressive increase in multinucleated fibers, as visualized by nuclei and cytoplasm fluorescence co-localization and longer cell fibers over time (Fig 1B). Comparison of cytoplasm and nucleus fluorescent signals showed a statistically significant higher contrast for cGFP+nmCherry than cmCherry+nGFP labelling (Fig 1C).

**Fig 2:**
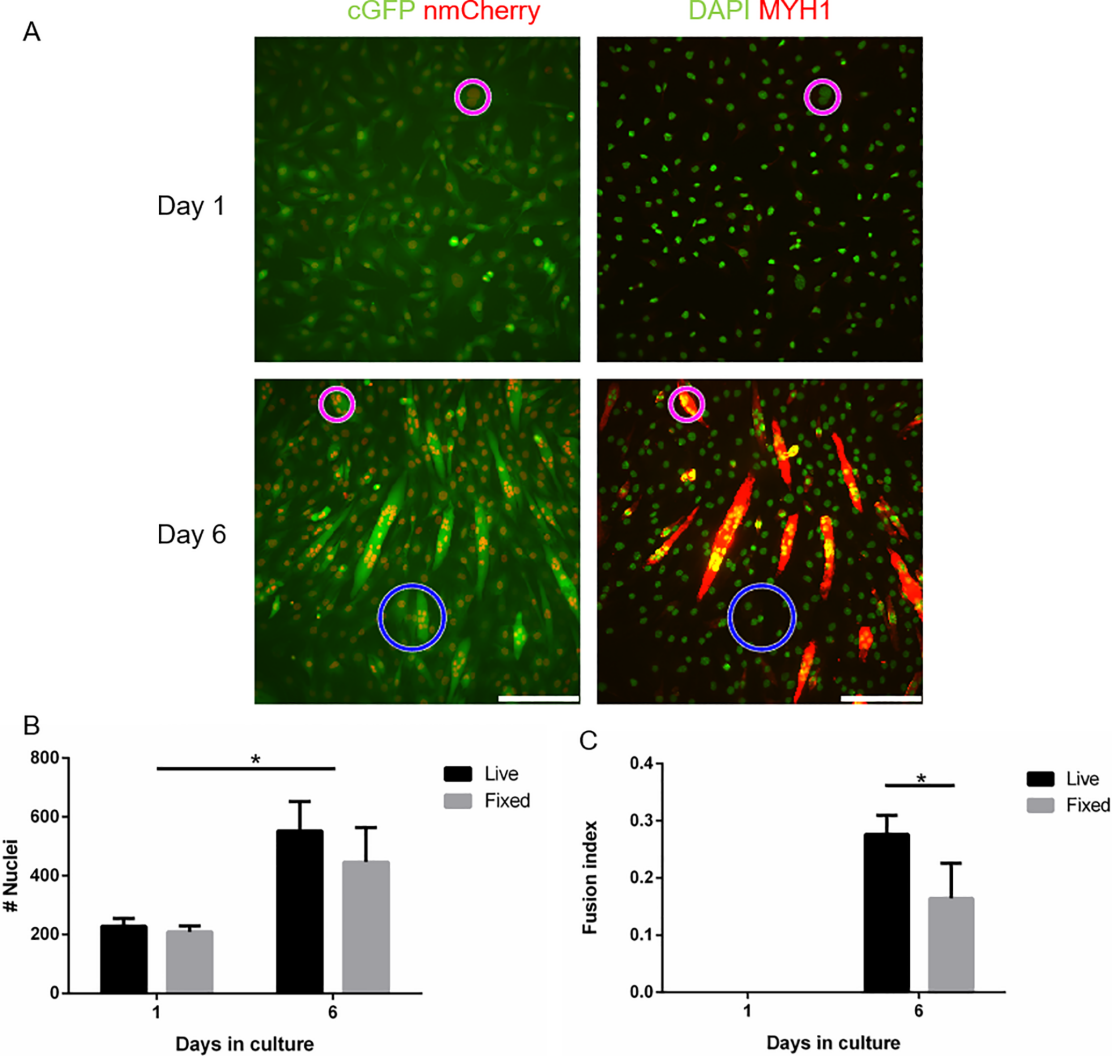
Immunostaining and live cell imaging of C2C12 fusion. (A) Representative images of cGFP (green) and nmCherry(red) in parallel with MyHC (red) and DAPI (green) immunostaining, showing differences between matched (magenta) and mismatch (blue) regions. (B,C) Calculated nuclei counts and fusion indices in both methods (scale bar 200**μ**m). * p<0.05.

To confirm the results of live cell fluorescent imaging, we compared them with immunostaining of fixed cells for myosin heavy chain (MYH) expression. Similarities were observed in myofiber formation (Fig 2A), and an increase in nuclei count and fusion was observed in both methods (Fig 2B and 2C). C2C12 fusion was greater in live cell imaging than in fixed cells (Fig 2B and 2C). The reduced fusion index in fixed cells was likely due to cell detachment caused by washing during the immunostaining procedure.

### Double fluorescent cell labeling to assess the effects of modulation of the myogenic process on myoblast fusion

**Fig 3:**
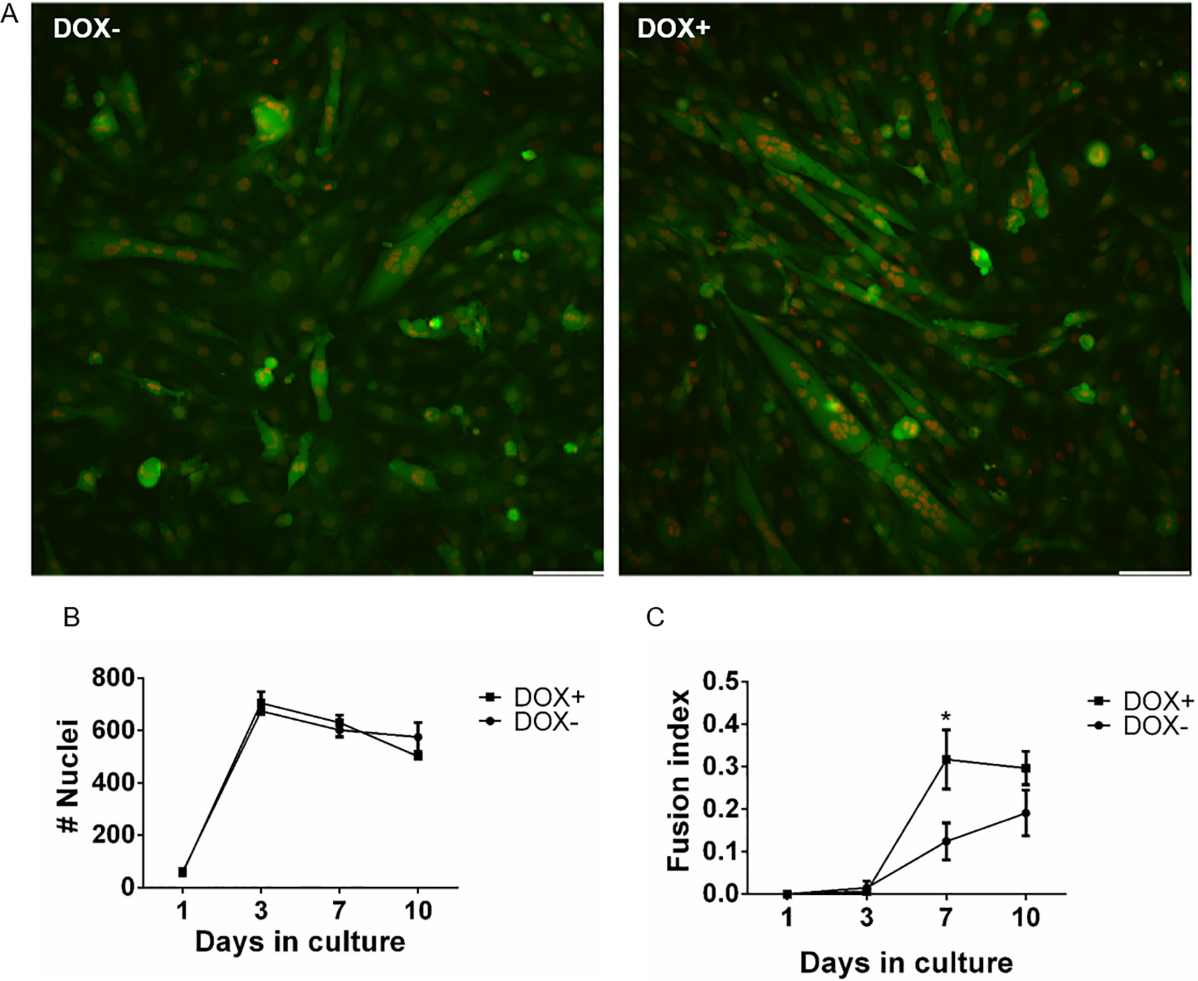
MYF5 overexpression induces myoblast differentiation. The effects of *Myf5* overexpression on C2C12 cell growth (B) and fusion (A,C) was determined using the double fluorescent cell labeling technique with images shown for day 7 (scale bar- 100**μ**m). *p<0.001.

First, the effect of *Myf5* overexpression on myoblast differentiation was examined using the double fluorescent cell labeling technique (Fig 3C). Double labeled C2C12 cells were engineered to overexpress *Myf5* in response to doxocyclin (DOX) exposure. A significantly higher fusion index was observed in the presence of DOX on day 7 (Fig 3B), while the nuclei count did not differ between cells in the presence or absence of DOX (Fig 3A), indicating that cell growth was similar. Monitoring of the fusion dynamically showed that cells exposed to DOX arrived quicker to their maximal fusion index (Fig 3B and S1 Movie).

**Fig 4:**
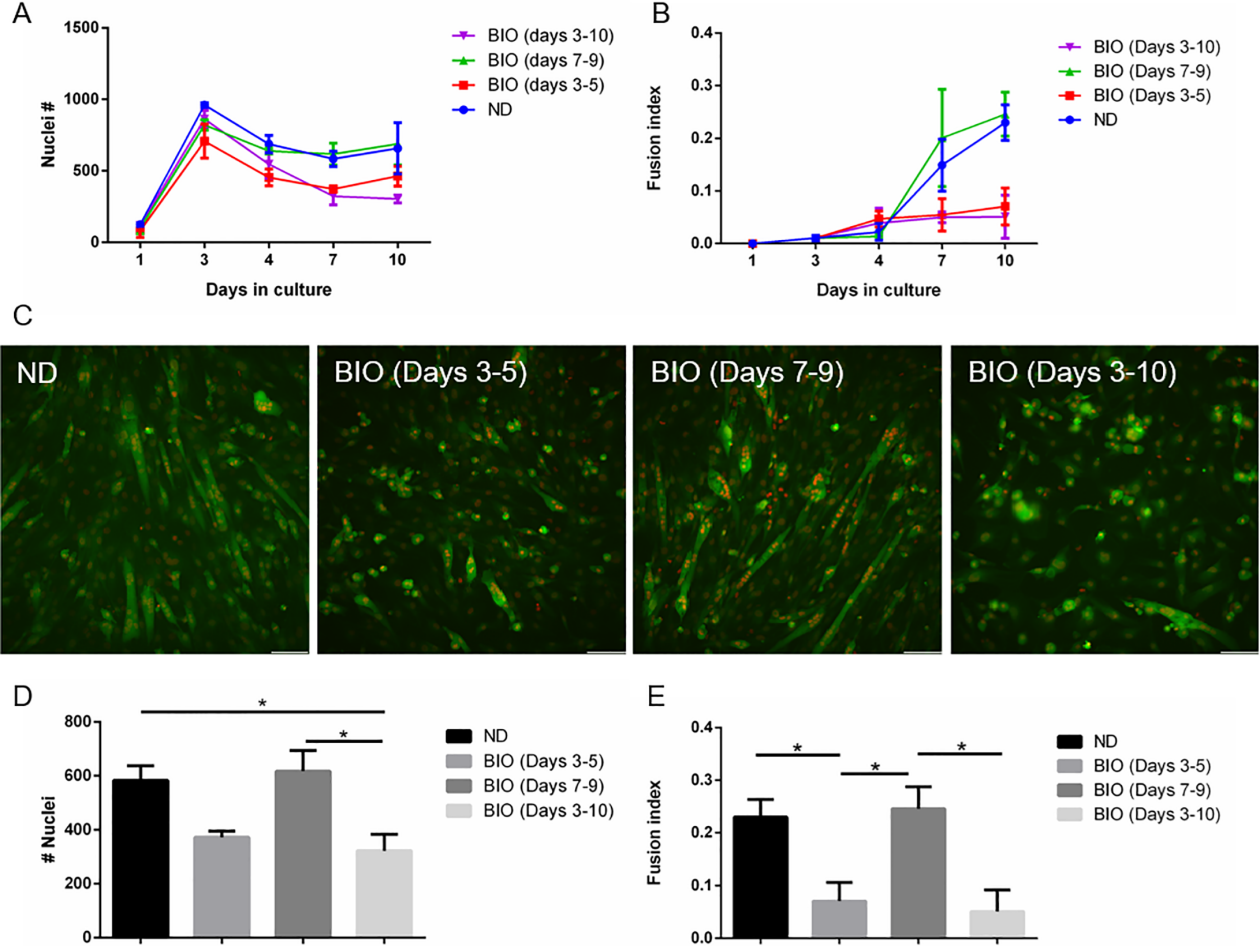
Activation of WNT signaling inhibits myoblast growth and differentiation. The effects of the Wnt agonist BIO on C2C12 cell growth in all days and specifically for day 10 (A,D) and fusion (B,C,E) was determined using the double fluorescent cell labeling technique with images shown for day 10 (scale bar- 100**μ**m). * p<0.05.

Activation of the WNT signaling pathway lead to premature or lack of myoblast differentiation [38,39]. We tested the effect of a WNT agonist, 6-bromoindirubin-3'-oxime (BIO), on C2C12 fusion by exposing cells to BIO at different times during the culture period (Table 1). Compared to cells incubated in the absence of BIO (ND) and during days 7–9, nuclei count and fusion indices, measured at day 10 (Fig 4D and 4E), were significantly lower for cells continuously exposed to BIO (days 3–10) and the days 3–5 exposure group. In addition, myotube size appeared smaller for these exposure periods compared with later exposure and control groups (Fig 4C).

**Fig 5:**
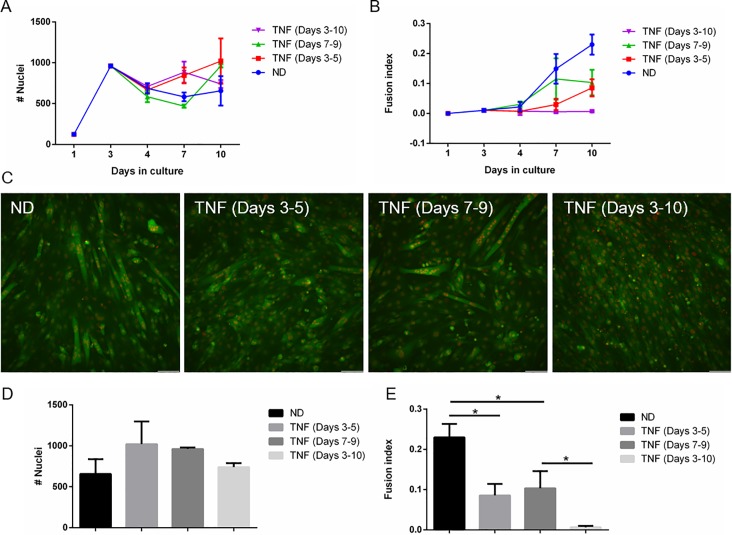
Exposure to TNFα inhibits myoblast differentiation but not growth. The effects of TNFα on C2C12 cell growth (A,D) and fusion (B,C,E) was determined using the double fluorescent cell labeling technique (scale bar- 100**μ**m). *p<0.05.

Tumor necrosis factor-alpha (TNFα) is involved in muscle regeneration and pathology [40,41]. Continuous exposure of C2C12 to TNFα and exposure at days 3–5 significantly reduced the fusion indices measured at day 7. At day 10, fusion indices of C2C12 exposed to TNFα in days 3–5 and 7–9 were similar and significantly higher than cells continuously exposed to TNFα, but significantly lower than cells that were not exposed to TNFα (Fig 5B and 5E). In fact, continuous exposure completely inhibited C2C12 fusion (Fig 5C and 5E). In contrast, TNFα had no significant impact on nuclei count (Fig 5A and 5D), while cell fusion was lower for all conditions exposed to TNFα.

### The effects of scaffold’s surface modifications on myoblast fusion determined by double fluorescent cell labeling

**Fig 6:**
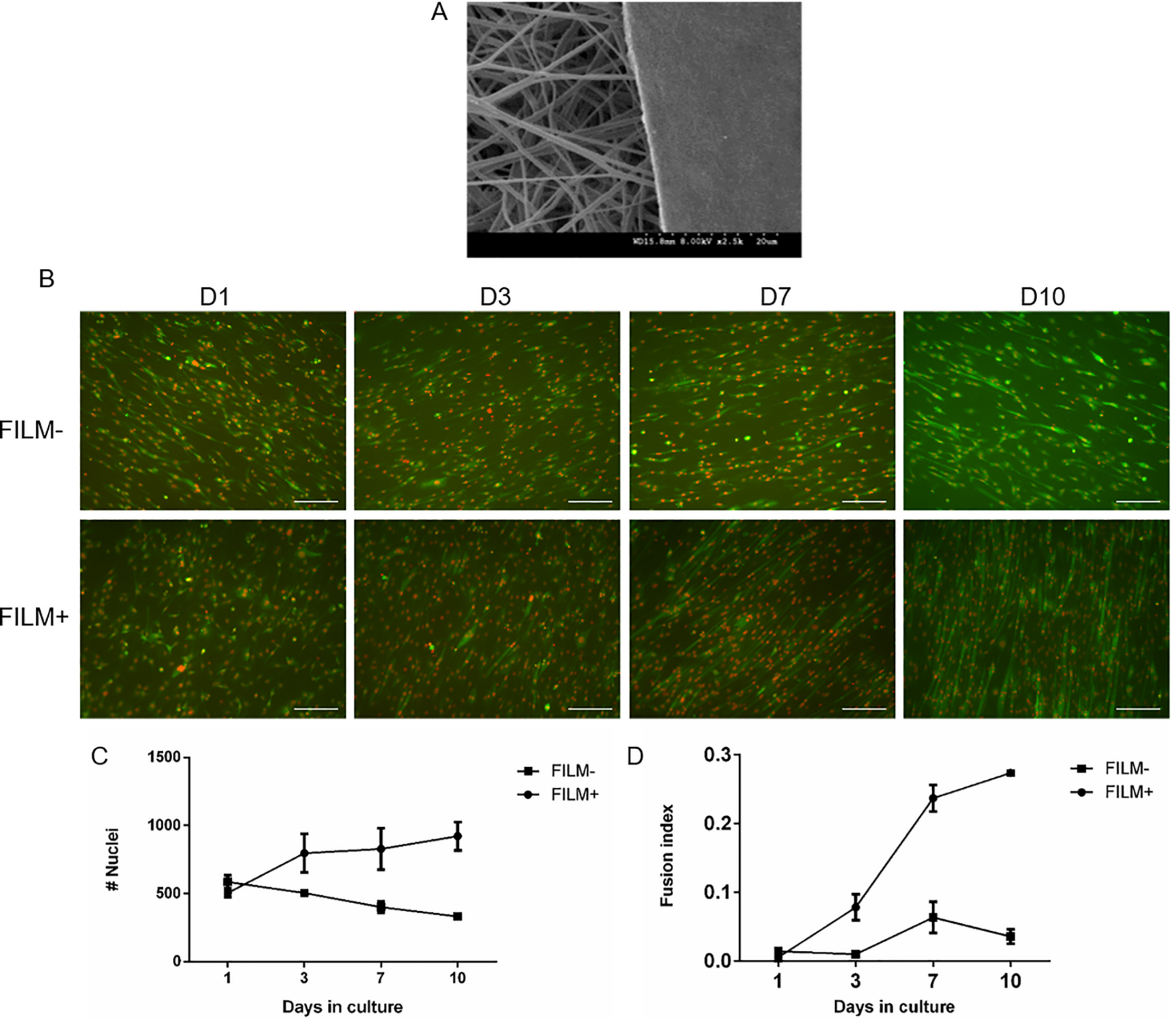
Collagen I coating of scaffolds improves myoblast growth and differentiation. (A) Scanning emission microscopy of electrospun scaffold coated with collagen I. The effects of Collagen I coating on C2C12 cell growth (C) and fusion (B,D) was determined using the double fluorescent cell labeling technique. (Scale bar-200**μ**m).

It is well documented that the growth of cells is greatly affected by the type of biomaterial they grow on and the surface topography of the scaffold [15,42,43]. However, biomaterial-based scaffolds for tissue engineering are usually opaque, which creates obstacles for direct imaging and monitoring. In addition, scaffolds containing ECM proteins, such as collagens, have high level of autofluorescence [44].We hypothesized that fluorescence imaging will facilitate imaging of cells seeded on opaque and autofluorescent biomaterials. Accordingly, the effects of collagen I coating of electrospun scaffolds (Fig 6A) was tested on C2C12 growth and fusion. Collagen I coating appeared to improve cell growth and cell fusion compared to uncoated scaffolds (Fig 6B–6D). The double fluorescent labeling enabled epifluorescent imaging of cells on biomaterial scaffold surface.

## Discussion

Immunostaining is one of the gold standards for assessing cellular functions. However, since immunostaining is based on sacrificial procedures, the ability to recognize the dynamic nature of biological processes is limited to only a single time point for each sample. To overcome hurdles in studying myogenesis, other tools have been designed to assess myoblast differentiation in real-time based on live cell monitoring. These tools include assessment of physical properties and function such as contractility through displacement analysis [36], skeletal muscle thickness [45,46] and myoblast differentiation [46] through electrical impedance. Additionally, multiple imaging modalities have been applied to assess muscle biology, including bioluminescence imaging for luciferase tagged cell transplantation [47], second harmonic generation (SHG) imaging to quantify muscle striation patterns [48,49], MRI to assess oxidative phosphorylation in muscle [50], and multi-photon imaging for the assessment of myoblast oxidation level [51].

Live cell monitoring via fluorescent imaging is not cell destructive and permits detection of spectrally distinguished fluorophores specificity. Fluorescent-based live cell imaging has other advantages including the potential for high spatial and temporal resolution, and multiplexing capabilities including structural, genetic, and physical information. In the current study we present an imaging technique based on double fluorescent labeling of nuclei and cytoplasm in order to monitor and assess myoblast fusion in real-time. The labeling scheme permitted quantification of the number of nuclei and fusion indices with similar results as immunostaining. We determined specific fluorophore combinations to improve image contrast, and used the technique to determine the effects of myogenesis regulators MYF5, Wnt, and TNFα on myoblast fusion. We further used the technique to monitor cell fusion of myoblasts seeded on opaque scaffolds with different surfaces. Our results validate the use of double fluorescent labeling for assessment of myoblast differentiation, which can be further used for screening of drugs to improve muscle regeneration from injuries and for testing adverse effects of drugs on skeletal muscles.

Fluorescent protein tagging of cells has been used in a variety of cell monitoring applications such as monitoring protein dynamics, localization, and DNA damage in mouse embryonic stem cells [52]. However, genetic manipulation of proteins in order to create fluorescent probes might alter their function. The advancement of new gene editing tools along with larger utilization of transgenic animal models where cells are labeled with fluorescent protein fusion [53] suggest that these tools hold significant promise for the development of therapeutic compounds to treat musculoskeletal injuries [36]. For the current study, we chose to use the C2C12 murine myoblast cell line that has been previously used in myogenesis research. Several fluorescent tagging tools have been developed to monitor C2C12 cell line myoblast differentiation (see Table 2 for a comparison between such tools and the double label approach using C2C12 cells). For example, split GFP technology identifies fusion between cells from two populations expressing two complementary components of GFP, such that only fusion of cells from both populations results in fluorescence [35]. Alternatively, GFP expression can be driven by muscle creatine kinase (MCK) expressed during differentiation [54]. The double labeling approach resembles *MyHC* and DAPI staining but without the need to sacrifice the samples to assess fusion. On the other hand, live cell imaging of nucleus and cytoplasm may not be as specific as MCK-GFP, which only fluoresce when myogenic gene is expressed similar to immunostaining, and thus requires morphological recognition of all myotubes. The nuclei labeling also enabled cell counts that represent cell proliferation rates, compared with sacrificial methods such as direct cell counting (e.g. DAPI) and mitochondrial activity (e.g. MTS).

**Table 2:** Comparison of live fluorescent labeling tools.

Criteria	Double label	Split fluorescent protein [35]	MCK promoter driven fluorescence [54]
**Quantitation**	Fusion index, nuclei count, and fibers size	Signal intensity and mixed fiber size	Fluorescence intensity, fiber size
**Minimal fusion events recognition**	Three cells (difficult to decipher two dividing cells from two nuclei fiber)	Only two cells from two populations	Two cells as long as fluorescence reporter is sufficiently strong
**Dynamic range**	From three cells to multi nucleated fiber	Limited to fluorescent intensity which may not change significantly with more fusion	Dependent on fluorescent intensity range
**Robustness**	Entire cell population	Only mixed fusion events are recognized	All myogenically differentiated cells recognized

### Image analysis and quantitation of cell fusion

Advances in image acquisition and live cell imaging have led to high throughput imaging platforms. Yet, automation of image analysis has limited accessibility for analysis of muscle phenotype [55,56]. While automated analysis for cell count and localization has been successful, automated analysis of complex structures, such as muscle fibers, is limited; this is partially due to the high density of fibers and high variance in fiber size and morphology. A high rate of false positives and/or false negatives prevent effective assessment, as fusion can be either over estimated or underestimated. Adaptation of automated analysis for fiber detection could reduce user error and increase sample sizes. In the current work, we tested two labeling schemes, which included tagging the cytoplasm and nucleus with green or red fluorescent protein that allowed for monitoring of progressing cell fusion over time. The label choice showed its importance for improving imaging sensitivity. As expected, the longer wavelength (mCherry compared with GFP) emission gave a better contrast for cell grown on culture plates. We based the analysis on a hybrid system: automated segmentation of cell nuclei with user corrections, and in parallel, larger structures were marked as fibers (Fig 1E), allowing for quantitative assessment of cell fusion. This analysis requires comprehensive user input, and as such it can be influenced by user error. Furthermore, the analysis was performed in one focal plane, which made it harder to detect multiple layers of cells. Developments in machine learning and computer vision have the potential for improving automated analysis of muscle fibers [57]. These types of developments along with the application of fast scanning 3D imaging would increase the utilization of high throughput acquisition tools.

The potential for noninvasive quantitation of muscle fusion will be advantageous for the detection of small changes in fusion level phenotype. In addition, since static culture of myoblasts on plastic has limited myotube stability [58], the dynamic monitoring of fusion can assist in determining relevant time points for fusion comparison. Such quantitation can complement information about tissue function [59] and cellular pathway activity [60]. Therefore, this assay can be used for optimization of muscle regeneration and drug screening.

### The potentials of fluorescent imaging for screening modulators of muscle regeneration

High throughput drug screening for skeletal muscle, like many other tissues, offers great promise when compared to animal models, allowing testing of multiple compounds and conditions with lower cost and shorter time [61]. Specifically, considering the dynamic nature of myoblast differentiation drug screening can assist in assessing the impact of timing when modulating myoblast response in different stages of differentiation. While several tools have been used to assess skeletal muscle tissue based on function in tissue engineered constructs[59], non-invasive quantitation of fusion level can provide better sensitivity to cell response correlating directly to cellular level response. In the current study, we have used the double labeling approach to determine overall fusion levels. Future adaptation to this approach can incorporate myogenic gene reporter-driven fluorescent proteins [54] during different stages of fusion in order to distinguish between early and later stages of myogenesis. We implemented the double fluorescence labeling techniques for assessing different modulators of myoblast differentiation. *Myf5* is a myogenic transcription factor expressed in the early stages of myogenesis [62]. As expected, *Myf5* overexpression increased fusion, which was significant by day 7. Future applications using tagged C2C12 may incorporate supportive patterns [58] and mechanical conditioning, which can promote prolonged tissue growth resembling more the physiological conditions faced by myoblasts in skeletal muscle tissue [63].

The WNT pathway is known to play a role in maintaining health of skeletal muscles and during disease [38,64]. Canonical Wnt pathway activation leads to cell cycle arrest and promotes the later stages of myogenesis [65]. On the other hand, our data show that prematurely arresting cell proliferation by exposing the cells to the Wnt agonist BIO at days 3–5 resulted in less cell fusion. In contrast, Wnt pathway activation at days 7–9 had a limited effect on cell fusion, suggesting that specific temporal regulation of the WNT pathway is necessary for optimal myogenesis.

TNFα, an important inflammatory factor, has a critical regulatory role in muscle regeneration. There are conflicting results for its specific activities; some studies showed that myogenic differentiation is blocked or delayed in the absence of TNFα signaling while others showed disruption of myoblasts differentiation and increased inflammatory activity which may correlate with its levels in cells [40,66,67]. Our results with continuous exposure of myoblasts to TNFα, which resembles chronic inflammation, completely inhibited cell fusion. In contrast, the nuclei count was not significantly changed, suggesting that the effect of TNF on fusion was not due to cytotoxicity.

Overall, the application of double fluorescent tagging in myoblasts can complement other screening platforms used for skeletal muscle biology. For example, 3D tissue constructs containing murine myoblasts from mdx mouse model for Douche dystrophy were assessed for contractile force using multiple compounds such as IGF-1, and creatine [68]. Double tagging such cells can add noninvasive cell level information in parallel to the contractile force measures. Another example of a screening assay relies on luciferase reporter for NF-κB activity [60]. While different compounds were assessed for their ability to inhibit TNFα influence on NF-κB pathway activity, the fusion phenotype assessment using double label fluorescent tagging can assist in recognizing if such condition can also recover myoblast fusion levels.

### Double labeled fluorescent imaging for monitoring bioengineered muscle constructs

One of the main motivations for fluorescent labeling of cells for tissue engineering purposes is to permit monitoring of cells seeded onto and within biomaterial scaffolds. The scaffolds have to support cellular growth and differentiation while maintaining mechanical integrity. However, many of these scaffolds are opaque and do not permit monitoring of cells via light microscopy. In our study we used elctrospinning technology to create scaffolds that offer better mechanical strength [69] but may not be optimal for efficient myoblast differentiation. To overcome this problem, the scaffolds were coated with a thin layer of collagen I, resulting in significant improvement of cell fusion. Data showed that collagen can support muscle cell growth and fusion, however collagen has strong autofluorescence [70]. The double fluorescent tagging allowed us to monitor both nuclei count and fusion levels over time on a collagen coated scaffold without the need for sacrificial staining. Similar applications are possible in a patterned substrate, which can allow for longer myotube stability *in vitro* [71].

## Conclusions

Double fluorescent labeling of cells in the nucleus and cytoplasm can facilitate imaging and quantification of myogenesis in real-time. This labeling scheme resulted in sufficient contrast in an otherwise high auto-fluorescence background due to the high scattering nature of the biomaterial scaffold. Live cell imaging results were comparable to immunostaining, providing good quantitation of cell growth and fusion. The double fluorescent labeling may be used to screen and test modulators of muscle regeneration as well as monitoring cell growth and differentiation in opaque scaffolds.

## Supporting information

S1 Movie Real time imaging of double labeled C2C12. cGFP nmCherry C2C12 cells expressing Tet-on *MYF5* were incubated in the presence and the absence of DOX (2 μg/ml) and images were recorded every 2 hours for 7 days.
(MOV)
